# Morphological changes of the anterior alveolar bone due to retraction of anterior teeth: a retrospective study

**DOI:** 10.1186/s13005-021-00277-z

**Published:** 2021-07-16

**Authors:** Qiannan Sun, Wenhsuan Lu, Yunfan Zhang, Liying Peng, Si Chen, Bing Han

**Affiliations:** Department of Orthodontics, Peking University School and Hospital of Stomatology & National Center of Stomatology & National Clinical Research Center for Oral Diseases & National Engineering Laboratory for Digital and Material Technology of Stomatology & Beijing Key Laboratory of Digital Stomatology & Research Center of Engineering and Technology for Computerized Dentistry Ministry of Health & NMPA Key Laboratory for Dental Materials, 22 Zhongguancun South Avenue, Haidian District, Beijing, 100081 People’s Republic of China

**Keywords:** Alveolar bone morphology, Incisor retraction, Orthodontic extraction treatment, Point A, Point B

## Abstract

**Backgroud:**

To analyze the morphological changes of the anterior alveolar bone after the retraction of incisors in premolar extraction cases and the relationship between incisor retraction and remodeling of the alveolar base represented by points A and B displacements.

**Methods:**

Pre- (T0) and post-treatment (T1) lateral cephalograms of 308 subjects in the maxilla and 154 subjects in the mandible who underwent the orthodontic treatment with extraction of 2 premolars in upper or lower arches were included. Alveolar bone width and height in both the maxillary and mandible incisor area were measured at T0 and T1 respectively. By superimposing the T0 and T1 cephalometric tracings, changes of points A and B, and the movement of the incisors were also measured. Then the correlation between incisor movement and the displacements of points A and B was analyzed.

**Results:**

The alveolar bone width (ABW) showed a significant decrease in both maxilla and mandible (*P* < 0.001) except the labial side of the mandible (*P* > 0.05). The alveolar bone height (ABH) showed a significant increase in the labial side of maxilla and a significant decrease in the lingual side of maxilla and mandible. A strong positive correlation was verified between incisor movement and position changes of points A and B in both horizontal and vertical directions.

**Conclusions:**

Anterior alveolar bone width and height generally decreased after orthodontic treatment. Incisor retraction led to significant position changes of points A and B. The decrease of anterior alveolar bone due to significant incisor retraction should be taken into account in treatment planning.

## Background

Tooth extraction is a common dental procedure intreating crowding and protrusion. The extraction space is used for retraction of the anterior teeth accompanied by remodeling of the alveolar bone, thus aligning the dentition and reducing facial convexity [1]. The extent of alveolar retraction resulting from orthodontic treatment should be directly related to the change after the final treatment [2]. Moreover, with the advancements in techniques, particularly the widespread use of implant anchorage, the indications of orthodontic treatment have expanded. Therefore, the extent of tooth movement has remarkably expanded and good outcomes are achieved in cases with various complex dentomaxillofacial deformities [3, 4]. However, it is unclear whether alveolar bone remodeling that occurs during orthodontic treatment always follows the direction and extent of tooth movement.

In orthodontic tooth movement, force induces alveolar bone resorption on the pressure side and bone formation on the tension side [5]. A classical theory in orthodontics is that the alveolar bone follows the direction of tooth movement and the width of alveolar bone remains unchanged [6]. However, many studies which assessed the periodontal status after orthodontic treatment have reported that excessive retraction of the anterior teeth may lead to iatrogenic sequelae such as alveolar bone loss, dehiscence, fenestration, and gingival recession [7–11]. Therefore, it’s important to verify the true capability for bone remodeling in alveolar bone to avoid the unwanted side-effects. Previous studies evaluating the relationship between incisor retraction and alveolar bone width/height generally included a small sample size, which may lead to more biasof the conclusions. Therefore, a more detailed study with a large sample size should be conducted to investigate the changes of alveolar bone.

Point A, where the lower front edge of the anterior nasal spine meets the front wall of the maxillary alveolar process and point B, the most posterior point on the anterior surface of mandibular symphysis are commonly used as indicators of the sagittal relationship between the maxilla and mandible in many analyses [12, 13]. However, the two anatomic landmarks are affected by the anterior alveolar bone remodeling with growth and orthodontic treatment [14, 15]. A few studies have attempted to investigate the effect of incisor inclination on the position of points A and B. Erverdi [15] showed a direct correlation between incisor inclination changes and point A. He found that point A and rotation point of incisor are positively correlated. Hassan et al. [16] found that if the upper incisor is retroclined by 10°, point A will move superiorly by 0.6 mm. In another study by Bicakci et al. [17], it was found that the proclination of the maxillary incisors (17.33°) along with the backward movement of the incisor root apex (2.12 mm) causes 1.04 mm backward movement of point A.. It was suggested to use the linear movement of the incisor apex, rather than the angular measurements to evaluate the sagittal change of point A [17]. Nevertheless, research on this topic has been limited thus far. Small sample size of the previous studies had decreased the statistical power of these studies [15–17]. In addition, to our knowledge, there is no study to evaluate the relationship between incisor retraction and point A movement.

In the present study, a large sample was used to study the influence of anterior tooth retraction on the position changes of both point A and point B, which is more comprehensive. We aimed to evaluate the changes in anterior alveolar bone width (ABW) and height (ABH) after incisor retraction and investigate the relationship between tooth movement and the position changes of points A and B. To avoid iatrogenic bone loss during treatment, it is important to understand the alveolar bone remodeling ability before orthodontic treatment and not to move teeth excessively during orthodontic treatment. The present study can provide a reference for the orthodontic treatment plan to establish the amount of tooth movement.

## Materials and methods

The study sample was selected from a database comprising over 11,000 patients who completed orthodontic treatment between 1997 and 2005 at Peking University School and Hospital of Stomatology. All the records were anonymized and de-identified prior to analysis. This retrospective cephalometric study was approved by the Institutional Ethics Committee (NO.: PKUSSIRB-201626016).

The inclusion and exclusion criteria were as follows: (1) Han Chinese ethnicity; (2) fixed-appliance orthodontic treatment with extraction of 2 premolars in upper and/or lower arches, respectively; (3) mean distance of the lingual movements of point UIE and LIE were more than 3 mm in maxilla and mandible, respectively; (4) 27° < sella-nasion-mandibular plane < 37°; (5) availability of the pre- and post-treatment lateral cephalograms which were of sufficient quality for identifying the relevant landmarks, all taken by the same X-ray machine; (6) lack of any significant medical history; (7) no craniofacial congenital malformation such as cleft lip and palate and syndromic disease; and (8) no need for orthognathic surgery.

Previous study has shown that anchorage loss is similar between extractions of first or second premolars [18], so the inclusion and exclusion criteria did not separate which premolar is extracted.

In the maxilla, pre-treatment (T0) and post-treatment (T1) cephalograms of 308 individuals (133 class I and 175 class II/1, 207 females and 101 males, mean treatment duration = 30.15 months) who met the selection criteria were included in this study. The patients ranged in age from 11 to 17 years, with an average age of 12.79 years.

In the mandible, 154subjects (82 class I and 72 class II/1, 103 females and 51 males, mean treatment duration = 30.31 months) aged from 11 to 17 years (mean age 12.82 years) qualified for the retrospective analysis.

The treatment protocol was standardized using an MBT (McLaughlin, Bennett, Trevisi) pre-adjusted appliance (Hangzhou Shinye Orthodontic Products; Hangzhou, China) with 0.022-in. slots. Initial levelling and alignment were performed with heat-activated round nickel titanium wires. Space closure was performed using rectangular 0.019 × 0.025-in. stainless-steel wire as working wire. Conventional anchorage such as TPA and/or headgear and elastic chains was used. Maximum anchorage mechanics were planned for all patients and all patients were experienced space closure with sliding mechanics and light forces. Cephalograms were taken before treatment (T0) and immediately after treatment (T1).

### Cephalometric analysis

**Fig. 1:**
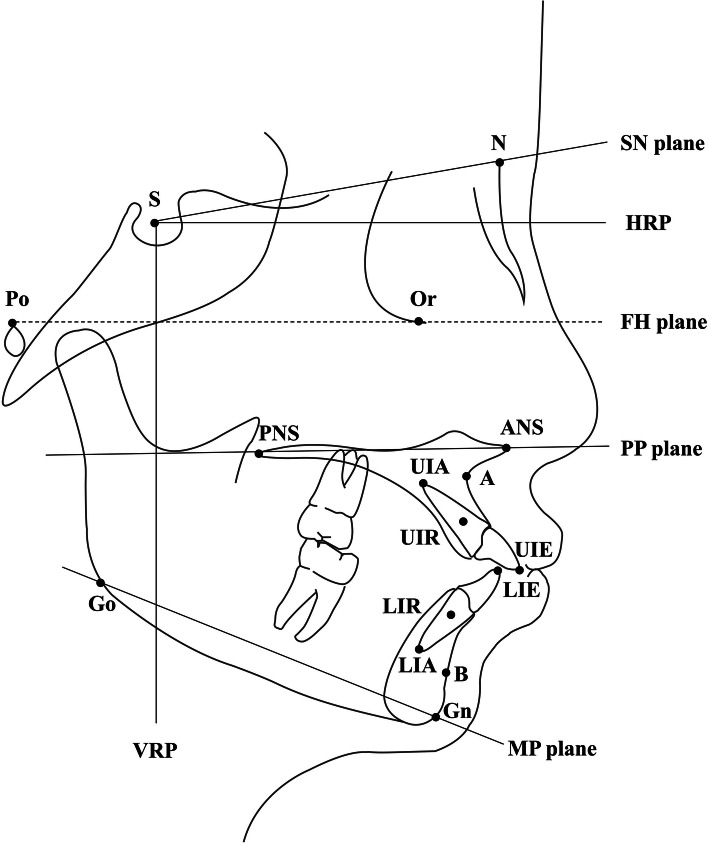
Illustration of cephalometric landmarks and reference planes

To control for magnification, all lateral cephalograms were taken with the same cephalostat with the consistent object-film distance. After the cephalograms were scanned, cephalometric landmarks were located three times each by three senior residents who had undergone calibration training and were blinded to the study objectives. The points with higher dispersion were automatically detected by a customized software and were checked by the same resident. The average of the nine locations of each landmark was used in subsequent calculations by the customized cephalometric software CIS (developed by the Department of Computer Science and technology of Peking University). The cephalometric landmarks and reference planes are shown and explained in Fig. 1 and Table 1 (the end of the document text file).

**Table 1:** Cephalometric landmarks and reference plane measurements

Landmark/Plane	Abbreviation	Definition
Sella	S	The center of the pituitary fossa of the sphenoid bone.
Nasion	N	The junction of the frontonasal suture at the most posterior point on the curve at the bridge of the nose.
Porion	Po	The most superior point of the external auditory meatus.
Orbitale	Or	The lowest point on the average of the right and left borders of the bony orbit.
Posterior nasal spine	PNS	The tip of the posterior nasal spine.
Anterior nasal spine	ANS	The tip of the anterior nasal spine.
Point A	A	The most posterior point on the curve of the maxilla between the anterior nasal spine and superdentale.
Upper incisor edge	UIE	The incisal edge point of the most prominent upper central incisor.
Upper incisor apex	UIA	The incisal apex of the most prominent upper central incisor.
Upper incisor resistance	UIR	The intersection point of the root axis and the upper border of the cervical third of the root.
Point B	B	The most posterior point to a line from infradentale to pogonion on the anterior surface of the symphyseal outline of the mandible.
Lower incisor edge	LIE	The incisal edge point of the most prominent lower central incisor.
Lower incisor apex	LIA	The incisal apex of the most prominent lower central incisor.
Lower incisor resistence	LIR	The intersection point of the root axis and the lower border of the cervical third of the root.
Gonion	Go	The bisector of the angle between tangent through the posterior margin of the ascending ramus and tangent to the mandibular base at menton.
Gnathion	Gn	The most anterior-inferior point on the contour of the bony chin symphysis.
Sella-Nasion plane	SN	The plane through sella and nasion.
Frankfort plane	FH	The plane through porion and orbitale.
Palatal plane	PP	The plane through ANS and PNS.
Mandibular plane	MP	The plane through gonion and menton.
Horizontal reference plane	HRP	The plane parallel to FH plane passing through sella.
Vertical reference plane	VRP	The plane was drawn as a perpendicular to HRP at sella.

**Fig. 2:**
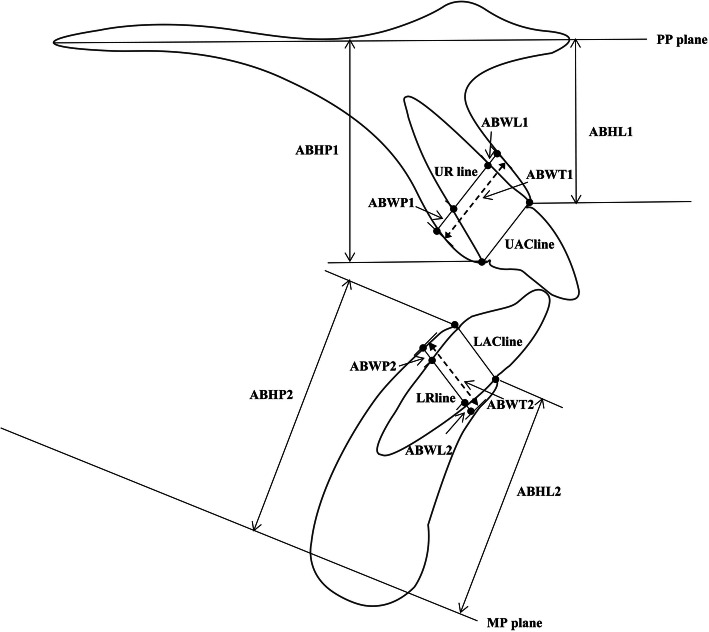
Alveolar bone width and height measurements before and after treatment. UAC line: a line that connects the labial and palatal alveolar crest (AC) points of upper incisor; UR line: a line parallel with UAC line passing through the center of resistance of the upper incisor (UIR); ABWL1, ABWP1, and ABWT1: labial, palatal, and total alveolar bone width of the upper incisor, respectively; LAC line: a line that connects the AC points of the lower incisor; LR line: a line parallel to LAC line passing through the center of resistance of the lower incisor (LIR); ABWL2, ABWP2, and ABWT2: labial, lingual, and total alveolar bone width of the lower incisor, respectively; ABHL1 and ABHP1:labial and palatal alveolar bone height of the upper incisor, respectively; ABHL2 and ABHP2:labial and palatal alveolar bone height of the upper incisor, respectively

The ABW of the labial, palatal/lingual and total alveolar crest were determined at the level of the center of resistance of the central incisors, which in this study was defined as a point located on the long axis of the tooth at a distance of 1/3 of the root length when measured from the alveolar crest (Fig. 2), [19]. UIR and LIR were used to represent the center of resistance of the central incisors in the maxilla and mandible respectively. A line passing through the center of resistance and parallel with the AC line (a line that connects the labial and palatal/lingual alveolar crest points) was used as the reference line (the observed level for alveolar bone width measurements). To ensure the consistency of the observed level, the distance between the AC line and the reference line on the pre-treatment cephalogram was recorded and then transferred to the post-treatment cephalogram. At this level, the labial (anterior), palatal/lingual (posterior), and total alveolar bone width was assessed in the maxilla (ABWL1, ABWP1, and ABWT1) and mandible (ABWL2, ABWP2, and ABWT2) at T0 and T1 respectively.

UAC line: a line that connects the labial and palatal alveolar crest (AC) points of upper incisor; UR line: a line parallel with UAC line passing through the center of resistance of the upper incisor (UIR); ABWL1, ABWP1, and ABWT1: labial, palatal, and total alveolar bone width of the upper incisor, respectively; LAC line: a line that connects the AC points of the lower incisor; LR line: a line parallel to LAC line passing through the center of resistance of the lower incisor (LIR); ABWL2, ABWP2, and ABWT2: labial, lingual, and total alveolar bone width of the lower incisor, respectively; ABHL1 and ABHP1:labial and palatal alveolar bone height of the upper incisor, respectively; ABHL2 and ABHP2:labial and palatal alveolar bone height of the upper incisor, respectively.

The ABH was measured as the vertical distance from both the labial and palatal/lingual side of the alveolar crest to the palatal plane in the maxilla (ABHL1 and ABHP1) and to the mandibular plane in the mandible (ABHL2 and ABHP2) (Fig. 2).

(a): Illustration of the superimposition of pre- and post-treatment tracings on the palatal plane at the ANS to determine the change in the position of point A.

(b): Illustration of the superimposition of pre- and post-treatment tracings on the mandibular plane at the gnathion to determine the change in the position of point B.

**Fig. 3:**
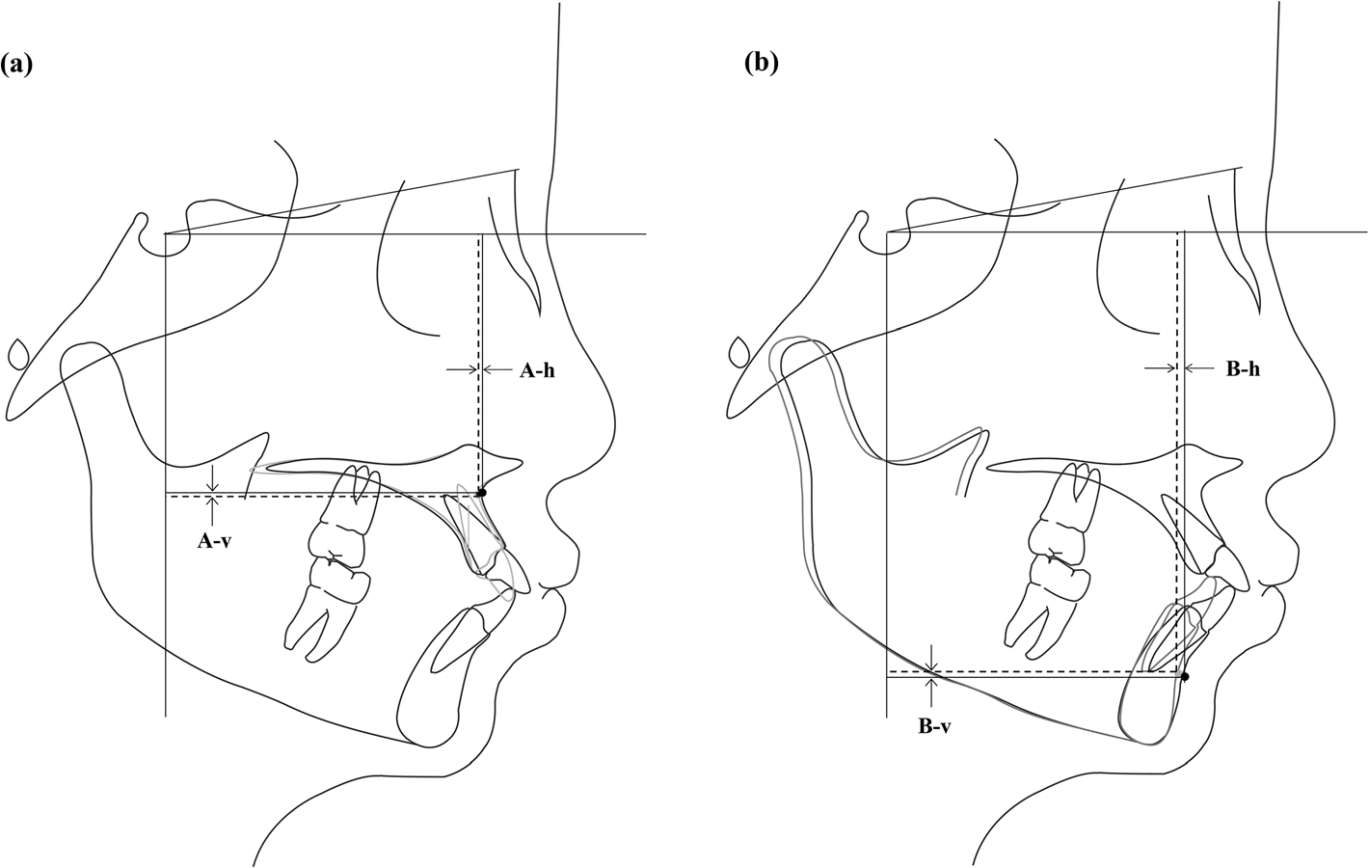
(**A**): Illustration of the superimposition of pre- and post-treatment tracings on the palatal plane at the ANS to determine the change in the position of point A. (**B**): Illustration of the superimposition of pre- and post-treatment tracings on the mandibular plane at the gnathion to determine the change in the position of point B.

The changes in the position of the upper incisor and point A were measured by superimposing the T0 and T1 lateral cephalograms on the palatal plane at the anterior nasal spine point (ANS) (Fig. 3A). On this superimposition, a horizontal line passing through the sella, parallel with the Frankfort plane, was drawn to form a horizontal reference line. A line perpendicular to the horizontal reference line, passing through the sella, was used as the vertical reference line. Three points on the most prominent upper central incisor- the incisal edge point (UIE), the center of resistance (UIR) and the apex of the root (UIA) were selected to be measured to reflect the position change of the upper incisor. The changes in the position of the lower central incisor and point B were measured by superimposing the T0 and T1 lateral cephalograms on the mandibular plane at the Gnathion (Fig. 3B). The antero-posterior and vertical changes in the position of the lower incisor and point B were determined using the same horizontal and vertical reference lines described above. LIE, LIR and LIA, the counterparts of UIE, UIR and UIA were used to reflect the position change of the lower incisor. Pre-treatment lateral cephalometric radiographs were traced with black lines, while post-treatment cephalograms were traced with gray lines.

### Statistical analysis

All measurements were conducted by two trained examiners. The intraclass correlation (ICC) was 0.96. The average measurements were used for analysis. Since the data showed a normal distribution, *t* tests were used. The paired t-test was used to evaluate the bony changes resulted from incisor retraction. The one-sample t-test was used to evaluate the changes in the position of the incisor and points A and B. Pearson’s correlation analysis was used to analyze the correlations between the amount of incisor movement and the position changes of points A and B. The statistical analyses were performed with SPSS 27.0 (IBM, Armonk, NY), with a significance level of 0.05.

## Results

The changes in ABW and ABH between T0 and T1 are shown in Table 2 and Fig.5A. In the maxilla, the labial, palatal and total alveolar bone width all decreased significantly after incisor retraction (*P* < 0.05, *P* < 0.001, and *P* < 0.001, respectively). Furthermore, the palatal side exhibited significant greater bone width reduction than the labial side (palatal side: − 0.27 ± 0.88 mm,*P* < 0.001;labial side: − 0.07 ± 0.47 mm, *P* < 0.05). In the mandible, the same decrease trend was found in both lingual and total alveolar bone width measurements (*P* < 0.001). The labial bone width of mandible exhibited slight decrease, though statistically insignificant (*P* > 0.05). The labial side of ABH showed a significant increase in the maxilla and a significant decrease in the mandible(*P* < 0.001 and *P* < 0.01, respectively); the lingual side of ABH showed a significant decrease in mandible and maxilla.

**Table 2:** Changes of the anterior alveolar bone width and height before (T0) and after (T1) orthodontic treatment

Alveolar bone	T0	T1	T1-T0		N
	Mean ± SD (mm)	Mean ± SD (mm)	Mean ± SD (mm)	*P* value	
ABWL1	1.81 ± 0.39	1.75 ± 0.40	−0.07 ± 0.47	.012*	308
ABWP1	3.24 ± 0.79	2.97 ± 0.80	−0.27 ± 0.88	.000***	308
ABWT1	11.69 ± 1.26	10.71 ± 1.35	− 0.98 ± 1.11	.000***	308
ABHL1	17.70 ± 2.05	18.82 ± 2.34	1.12 ± 1.23	.000***	308
ABHP1	21.52 ± 1.97	21.24 ± 2.16	−0.28 ± 1.22	.000***	308
ABWL2	1.77 ± 0.46	1.76 ± 0.43	−0.01 ± 0.46	.811	154
ABWP2	1.88 ± 0.43	1.51 ± 0.35	−0.37 ± 0.43	.000***	154
ABWT2	8.62 ± 0.87	7.76 ± 0.80	−0.87 ± 0.75	.000***	154
ABHL2	29.05 ± 2.81	28.63 ± 3.35	−0.42 ± 1.74	.003**	154
ABHP2	29.75 ± 2.87	28.60 ± 3.26	−1.15 ± 1.50	.000***	154

†ABWL1, ABWP1, and ABWT1: labial, palatal, and total alveolar width of the upper incisor, respectively; ABWL2, ABWP2, and ABWT2: labial, lingual, and total alveolar width of the lower incisor, respectively. ABHL1 and ABHP1: labial and palatal alveolar bone height of the upper incisor, respectively; ABHL2 and ABHP2: labial and palatal alveolar bone height of the lower incisor, respectively.

‡* *P* < 0.05; ** *P* < 0.01; *** *P* < 0.001.

**Fig. 4:**
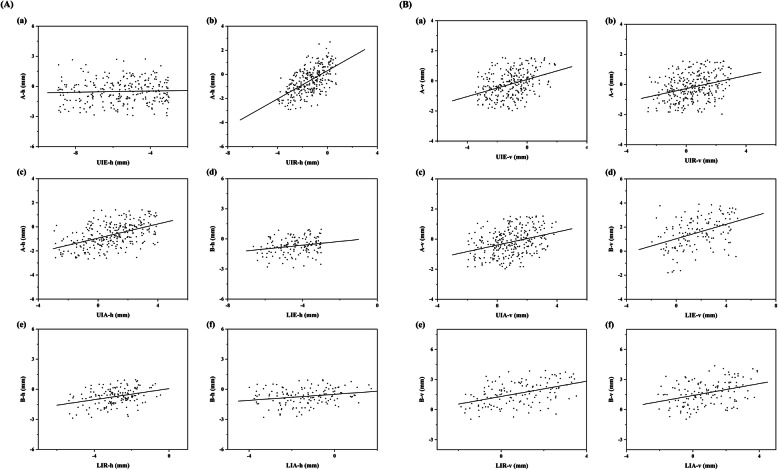
(**A**): Scatterplot of the sagittal position changes for the incisor and points A and B. (**B**): Scatterplot of the vertical position changes for the incisor and points A and B

The average changes of the points measured in this study are shown in Table 3. In sagittal direction, significant differences were observed at points UIE, UIR, UIA, A, LIE, LIR, LIA, and B after treatment. In the maxilla, the mean distance of the lingual movements of point UIE and UIR were 6.21 ± 2.25 mm and 1.60 ± 1.43 mm, whereas the mean distance of the labial movement of point UIA was 1.05 ± 2.10 mm. In the mandible, points LIE, LIR and LIA all moved backwards by an average distance of 4.65 ± 1.28 mm, 2.97 ± 1.22 mm and 1.52 ± 1.58 mm, respectively. In vertical direction, there were statistical differences for the displacements of all points. Point UIE moved downward, whereas points UIR and UIA moved in the opposite direction. Besides, the lower incisor showed upward movement as a whole. As shown in Table 4, a significant positive correlation was present between point A and the apical point UIA and UIR (r = 0.652, *P* < 0.001; r = 0.694, *P* < 0.001) in the horizontal direction. The correlation coefficient between point A and points UIE, however, was also significant on the border(*P* < 0.01). The correlation coefficients between point B and points LIE, LIR and LIA were also significant. Furthermore, the relationship between the displacement of the teeth and the displacement of points A and B was linear (Fig.4).

**Table 3:** Treatment changes of points in the horizontal direction

Variable	Mean ± SD (mm)	*p*-value	N
UIE-h	−6.21 ± 2.25	.000***	308
UIR-h	−1.60 ± 1.43	.000***	308
UIA-h	1.05 ± 2.10	.000***	308
A-h	−0.63 ± 1.23	.000***	308
UIE-v	−1.22 ± 1.52	.000***	308
UIR-v	0.38 ± 1.37	.000***	308
UIA-v	0.83 ± 1.53	.000***	308
A-v	−0.23 ± 0.97	.000***	308
LIE-h	−4.65 ± 1.28	.000***	154
LIR-h	−2.97 ± 1.22	.000***	154
LIA-h	−1.52 ± 1.58	.000***	154
B-h	−0.80 ± 0.96	.000***	154
LIE-v	1.75 ± 2.08	.000***	154
LIR-v	0.84 ± 1.66	.000***	154
LIA-v	0.80 ± 1.84	.000***	154
B-v	1.75 ± 1.65	.000***	154
UTL	−0.93 ± 1.42	.000***	308
LTL	−1.11 ± 1.41	.000***	154

†h: horizontal displacement of points; v: vertical displacement of points; UTL: tooth length of the upper incisor; LTL: tooth length of the lower incisor.

‡ *** *P* < 0.001.

**Table 4:** Pearson correlation Coefficients and *P* Values Between incisors movement and the displacements of points A and B

Variable	coefficient	P value
A-h & UIE-h	0.158	.005**
A-h & UIR-h	0.694	.000***
A-h & UIA-h	0.652	.000***
A-v&UIE-v	0.400	.000***
A-v & UIR-v	0.416	.000***
A-v & UIA-v	0.446	.000***
B-h&LIE-h	0.312	.000***
B-h& LIR-h	0.466	.000***
B-h & LIA-h	0.416	.000***
B-v&LIE-v	0.473	.000***
B-v & LIR-v	0.594	.000***
B-v & LIA-v	0.576	.000***

†h: horizontal displacement of points; v: vertical displacement of points.

‡** *P* < 0.01; *** *P* < 0.001.

## Discussion

Cephalometric analysis based on lateral cephalograms has been a mature and widely used tool for the studies of craniofacial anatomical structures [20]. Since lateral cephalometric radiography was introduced in 1931 [21], studies have been widely conducted and cephalometric normative value has been accumulated, which provides useful information in orthodontic diagnosis. However, although CBCT provides more extensive information, it still cannot replace the widely used lateral cephalometric radiography due to studies of the normative value data are insufficient [20]. CBCT is likely to replace lateral cephalograms completely as the field progresses. However, the current two-dimensional normative reference value of lateral cephalograms is an important criterion for diagnosis.

On the other hand, statistical power increases with increased sample size. At present, CBCT is mainly used for the diagnosis and treatment of impacted teeth, cleft lip and palate and skeletal discrepancies requiring surgical intervention, etc. [22]. Therefore, the studies based on CBCT usually were conducted using a small sample size, which could affects the statistical power to a certain extent. Therefore, in this retrospective study, a large sample consisted of pre- and post- treatment lateral cephalograms from the existed data base was used to evaluate the change in alveolar bone morphology (width and height) and investigate whether the position of points A and B would be affected by bone remodeling related to incisor retraction. The large sample size and comprehensive measurements lead to a greater chance for this study to reflect the most possible changes occurred incisor retraction in the orthodontic treatment.

The anterior alveolar bone defines the boundary for the retraction of the anterior teeth in orthodontic treatment. Though theoretically bone remodeling occurs during tooth movement, it remains controversial whether the changes in the anterior alveolar bone always follow the direction and quantity of tooth movement. De Angelis [5] described the bending capacity of alveolar bone, which suggested that the alveolar bone retained its structural characteristics and size as it moves with coordinated apposition and resorption. However, this bending capacity wasn’t verified by other studies [23–25]. The apposition and resorption of bone are in a dynamic situation during tooth movement. Melsen [23] indicated that most resorption activity occurs at sites that undergo compression, and reduced activity occurs in the tension zone. Bimstein et al. [24] suggested that the amount (mainly the height) of buccal alveolar bone might increase as a result of orthodontic treatment that involves lingual positioning of procumbent mandibular permanent central incisors without intrusion. In contrast, Sarikaya et al. [25] reported bone width in the mandible and in the lingual side of the maxilla was significantly decreased after orthodontic treatment, whereas maxillary bone thickness labial to the incisors remained unchanged. Similar results were found by Vardimon et al. [26], Ahn et al. [27], and Thongudomporn et al. [28]. One study found that upper incisor inclination and intrusion changes may increase the degree of alveolar bone loss [29]. In the present study, after the anterior teeth were retracted by more than 3 mm, we found that the labial alveolar bone width showed a significant decrease in the maxilla and an insignificant decrease in the mandible; the lingual side of the alveolar bone showed a significant decrease in both maxilla and mandible. Our results are consistent with the studies which showed the alveolar bone width decrease after retraction of the anterior teeth and suggested that the bone apposition process was slower than the resorption process. Significant increase of ABH was found on the labial side of the maxilla. A possible reason for this change might be the extrusion of the upper incisor during the retraction. Decrease of ABH was found on the palatal side of maxilla and both sides of mandible, especially the lingual side. Similar result was found by Lund et al. [30], who reported alveolar bone height decrease of the front teeth in premolar extraction cases and the most significant decrease found on the palatal/lingual side. On the palatal side, ABH of mandible was decreased more than maxilla (1.15 ± 1.50 mm and 0.28 ± 1.22 mm,respectively). This may due to the narrower width of the alveolar bone in the mandible (palatal/lingual alveolar bone width at T0, Table 2), which would be more sensitive to the stress concentration around the cervical area from the controlled tipping movement of the lower incisor. The marginal thin layer of bone on the lingual side of the mandible was more vulnerable to disappear during bone remodeling procedure. That may be the reason for the greater decrease of mandibular alveolar bone. Both the alveolar bone width and height decreased the most on the lingual side of the lower incisor suggested that more attention should be given to this area to prevent excessive bone resorption in the treatment. Previous studies focused on the changes in facial profile during orthodontic treatment [31]. Now public are more aware of the importance of healthy treatment while paying attention to aesthetics. More attention should be paid to the movement of anterior teeth to avoid severe alveolar bone loss.

**Fig. 5:**
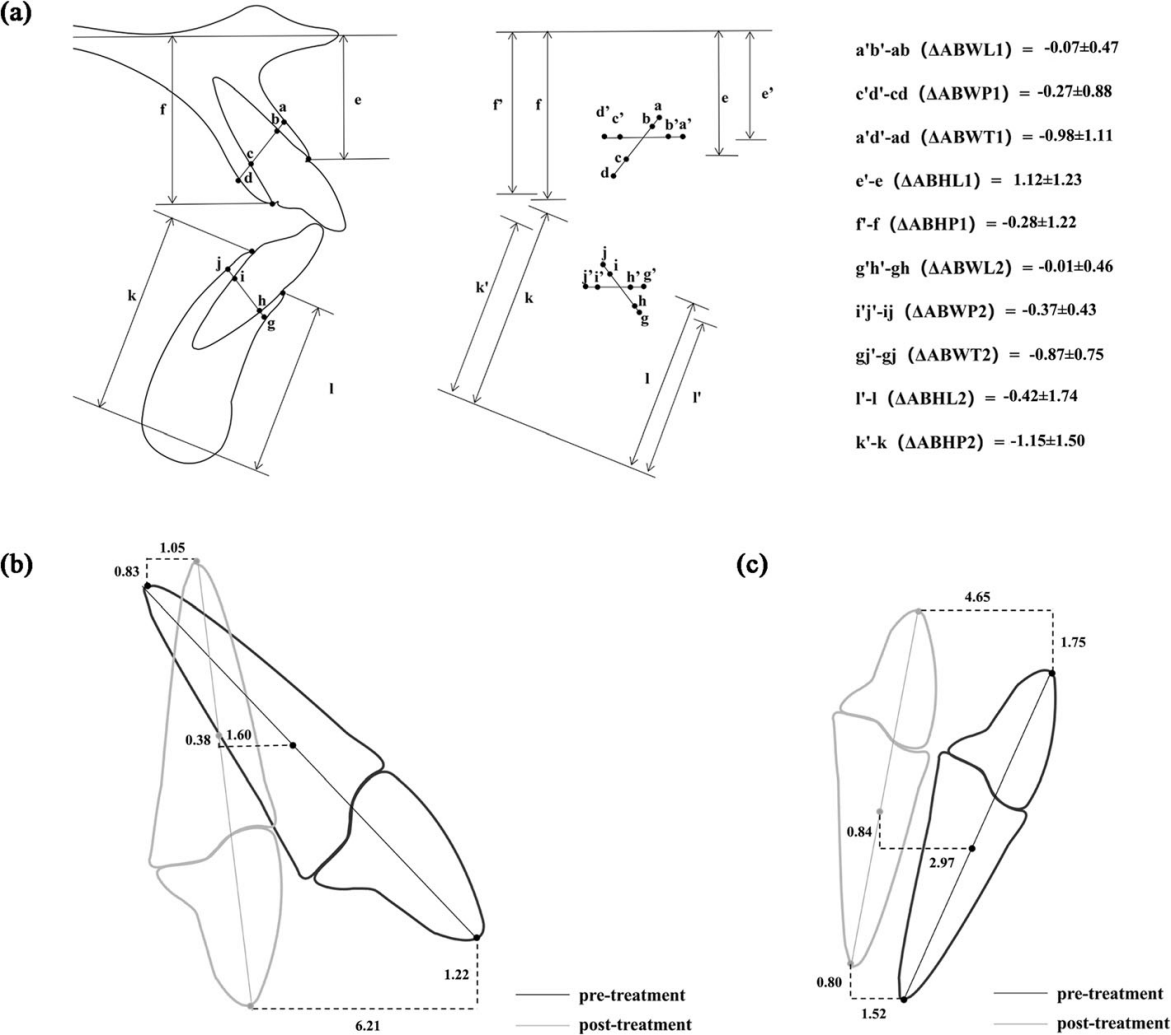
Illustration of changes of alveolar bone and tooth movements of incisors after orthodontic treatment. (**A**):Illustration of changes of the anterior alveolar bone width and height before (T0) and after (T1) orthodontic treatment. ab, cd, ad, e, f, gh, ij, gj, l and k:ABWL1, ABWP1, ABWT1, ABHL1, ABHP1, ABWL2, ABWP2, ABWT2, ABHL2 and ABHP2 before orthodontic treatment; a’b’, c’d’, a’d’, e’, f’, g’h’, i’j’, g’j’, l’, k’:ABWL1, ABWP1, ABWT1, ABHL1, ABHP1, ABWL2, ABWP2, ABWT2, ABHL2 and ABHP2 after orthodontic treatment;Δ:difference between post-treatment and pre-treatment. (**B**): Illustration of tooth movements of upper incisor after orthodontic treatment. (**C**): Illustration of tooth movements of lower incisor after orthodontic treatment.(unit: mm)

The upper incisor and lower incisor were found to move in different types in this study. UIE and UIR of the maxillary incisor moved lingually by 6.21 mm and 1.60 mm, respectively, whereas UIA moved labially by 1.05 mm. This result indicated that the incisor movement during retraction in the maxilla was mainly tipping, which meant that the edge and apex of the incisor moved in the opposite direction and the center of rotation located between the center of resistance and the apex (Fig.5B). The upper incisor became more upright during the retraction. In the mandible, LIE, LIR and LIA were found to move lingually by 4.65 mm, 2.97 m and 1.52 mm, which indicated the controlled tipping of lower incisors (Fig.5C). This finding is consistent with the results of Sarikaya et al. [25] and Vardimon et al. [26]. who observed that in patients undergoing retraction with torque, the result was combined movement with some tipping rather than pure translation.

Root resorption is a common side-effect of orthodontic treatment, especially with extensive tooth movement. In this study, the distance between UIE and UIA in maxilla and between LIE and LIA in mandible were measured as the length for upper and lower incisor respectively. The results showed that the length of the incisor decreased by 0.93 mm and 1.11 mm in maxilla and mandible respectively, which was close to the results of the meta-analysis conducted by Samandara et al. In their study, the average amount for root resorption in anterior teeth was found to be 0.9 mm [32]. Kaley and Phillips [33] indicated that the contact between the root and the cortical bone is an important cause for root resorption. Horiuchi et al. [34] reported that apex approximation to the palatal cortical plate due to incisor retraction was one of the critical factors for root resorption. In addition, insufficiency of the maxillary width during tooth movement could be a risk associated with root resorption [34]. One study observed the relationship between contact the incisive canal of upper central incisors and root resorption [35]. The results showed that contact between upper incisors and the cortical plate of the incisive canal cause significantly more apical root resorption than in the noncontact group. The result of this study also showed the decrease of the alveolar bone width after incisor retraction. Therefore, the alveolar width should be carefully evaluated before the treatment to prevent excessive incisor retraction which may lead to significant root resorption.

A few previous studies focused on the effect of incisal inclination changes on points A and B and did not consider changes caused by the sagittal and vertical movement of the incisor [15–17, 36, 37]. Al-Nimri et al. [37] stated that, in Class II division 2 malocclusion, the movement of point A, affected by local bone remodeling, occurred in a backward direction. An earlier study by Al-Abdwani [36] stated that each 10° proclination of the upper incisors resulted in a significant average change in point A of 0.4 mm in the horizontal plane. Moreover, each 10°proclination of the lower incisors resulted in a borderline significant average change in point B of 0.3 mm in the horizontal plane. Cangialosi and Meistrell [14] studied the effect of lingual root torque on the sagittal position of point A, and showed that the posterior movement of the apex of the maxillary incisors resulted in a 1.7-mm posterior movement of point A. Hassan et al. [16] reported that there was no evidence of significant horizontal and vertical displacement of point B due to lower incisor inclination changes. In the present study, we found that point A moved backward of 0.63 mm (*P* < 0.001) and downward of 0.23 mm (P<0.001) with the retraction of upper incisor. A positive correlation was observed between the position change of point A and the displacements of points UIE, UIR and UIA. In the mandible, point B showed significant movement both in sagittal and vertical direction. In addition, a positive correlation was found between the sagittal position of point B and the horizontal position changes of points LIE, LIR and LIA (r = 0.312, 0.466 and 0.416, respectively). The vertical displacement of point B and the displacements of points of lower incisors also showed a positive correlation (r = 0.473, 0.594 and 0.576, respectively). The result of Pearson correlation analysis indicated that the backward movement of points A and B increase with the extent of incisor retraction.

Although a large sample size was used, the limitations of this study should be considered. First, patients treated by different doctors with different treatment protocols may have different results. If all the samples were treated by one doctor, the consistency of the results would be good but less representative. Including more samples treated by different doctors will lead to a greater chance to find the general trend of the studied question. To reduce the effect of different treatment protocols on the result, the samples included in this study were all treated by fixed-appliance with extraction of 2 premolars in upper and/or lower arches and had more than 3 mm incisor retraction. Second, the center of resistance used in this study (UIR, LIR) was defined as a point located on the long axis of the tooth at a distance of 1/3 of the root length when measured from the alveolar crest [19]. It’s a well-defined point but would be affected by both the changes of the root length and the alveolar crest. To ensure the consistency of the measurement level, the pre-Tx reference line was transferred to the post-treatment cephalogram. Therefore, the UIR and LIR used in T1 ABW measurement were not the strictly defined one. We didn’t find a better way in which the true center of resistance could be used at the same time when the consistency of the pre- and post-treatment measurement level could be maintained. Finally, lacking of 3D images, we couldn’t know exactly to what extent incisors retraction will lead to iatrogenic sequelae such as dehiscence and fenestration. In our future researches, when the 3D sample size is sufficient, more details will be explored based on the results from this large sample size study serving as clues.

## Conclusions

Both the alveolar bone width and height decreased following anterior teeth retraction, which suggests that alveolar bone remodeling doesn’t always follows the direction and extent of orthodontic tooth movement. In most cases, the incisor retraction in orthodontic treatment was a combined movement with some tipping rather than pure translation. In addition, the position of anatomic landmarks point A and B could be affected by alveolar bone remodeling during orthodontic treatment. The capability of the anterior alveolar bone remodeling should be carefully analyzed in orthodontic treatment planning to avoid extensive incisor retraction and negative iatrogenic effects.

## Data Availability

The datasets used and/or analyzed during the current study are available from the corresponding author on reasonable request.

## Abbreviations

T0: Pre-treatment
T1: Post-treatment
ABW: The alveolar bone width
ABH: The alveolar bone height
AC line: A line that connects the labial and palatal /lingual alveolar crest points
ABWL1, ABWP1, and ABWT1: Labial, palatal, and total alveolar width of the upper incisor, respectively
ABWL2, ABWP2, and ABWT2: Labial, lingual, and total alveolar width of the lower incisor, respectively
ABHL1 and ABHP1: labial and palatal alveolar bone height of the upper incisor, respectively
ABHL2 and ABHP2: labial and palatal alveolar bone height of the lower incisor, respectively. The cephalometric landmarks and reference planes are explained in Table 1.

## References

[1] Diels RM, Kalra V, Deloach N, Powers M, Nelson SS. Changes in soft-tissue profile of African-Americans following extraction treatment. Angle Orthod. 1995;65(4):285–92. 10.1043/0003-3219(1995)065<0285:CISTPO>2.0.CO;2. 10.1043/0003-3219(1995)065<0285:CISTPO>2.0.CO;2 7486243

[2] Baek SH, Kim BH (2005). Determinants of successful treatment of bimaxillary protrusion: orthodontic treatment versus anterior segmental osteotomy. J Craniofac Surg.

[3] Alharbi F, Almuzian M, Bearn D (2019). Anchorage effectiveness of orthodontic miniscrews compared to headgear and transpalatal arches: a systematic review and meta-analysis. Acta Odontol Scand.

[4] Jing Y, Han X, Guo Y, Li J, Bai D (2013). Nonsurgical correction of a class III malocclusion in an adult by miniscrew-assisted mandibular dentition distalization. Am J Orthod Dentofac Orthop.

[5] DeAngelis V (1970). Observations on the response of alveolar bone to orthodontic force. Am J Orthod.

[6] Edwards JG (1976). A study of the anterior portion of the palate as it relates to orthodontic therapy. Am J Orthod.

[7] Wehrbein H, Bauer W, Diedrich P (1996). Mandibular incisors alveolar bone, and symphysis after orthodontic treatment. A retrospective study. Am J Orthod Dentofac Orthop.

[8] Wehrbein H, Fuhrmann RA, Diedrich PR (1994). Periodontal conditions after facial root tipping and palatal root torque of incisors. Am J Orthod Dentofac Orthop.

[9] Wainwright WM (1973). Faciolingual tooth movement - its influence on root and cortical plate. Am J Orthod.

[10] Joss-Vassalli I, Grebenstein C, Topouzelis N, Sculean A, Katsaros C (2010). Orthodontic therapy and gingival recession: a systematic review. Orthod Craniofac Res.

[11] Yared KF, Zenobio EG, Pacheco W (2006). Periodontal status of mandibular central incisors after orthodontic proclination in adults. Am J Orthod Dentofacial Orthop.

[12] Mills JR (1978). The effects of orthodontic treatment on the skeletal pattern. Br J Orthod.

[13] Seselj M, Duren DL, Sherwood RJ (2015). Heritability of the human craniofacial complex. Anat Rec (Hoboken).

[14] Cangialosi TJ, Meistrell ME (1982). A cephalometric evaluation of hard- and soft-tissue changes during the third stage of Begg treatment. Am J Orthod.

[15] Erverdi N (1991). A cephalometric study of changes in point a under the influence of upper incisor inclinations. J Nihon Univ Sch Dent.

[16] Hassan S, Shaikh A, Fida M (2015). Effect of incisor inclination changes on Cephalometric points a and B. J Ayub Med Coll Abbottabad.

[17] Bicakci AA, Cankaya OS, Mertoglu S, Yilmaz N, Altan BK (2013). Does proclination of maxillary incisors really affect the sagittal position of point a?. Angle Orthod.

[18] Steyn CL, du Preez RJ, Harris AM (1997). Differential premolar extractions. Am J Orthod Dentofac Orthop.

[19] Burstone CJ, Every TW, Pryputniewicz RJ (1982). Holographic measurement of incisor extrusion. Am J Orthod.

[20] Jung PK, Lee GC, Moon CH (2015). Comparison of cone-beam computed tomography cephalometric measurements using a midsagittal projection and conventional two-dimensional cephalometric measurements. Korean J Orthod.

[21] Broadbent BH (1931). A new x-ray technique and its application to orthodontia. Angle Orthod.

[22] Kapila SD, Nervina JM. CBCT in orthodontics: assessment of treatment outcomes and indications for its use. Dentomaxillofac Rad. 2015;44(1):20140282. 10.1259/dmfr.20140282 PMC4277443 25358833

[23] Melsen B. Biological reaction of alveolar bone to orthodontic tooth movement. Angle Orthod. 1999;69(2):151–8. 10.1043/0003-3219(1999)069<0151:BROABT>2.3.CO;2. 10.1043/0003-3219(1999)069<0151:BROABT>2.3.CO;2 10227556

[24] Bimstein E, Crevoisier RA, King DL (1990). Changes in the morphology of the Buccal alveolar bone of protruded mandibular permanent incisors secondary to orthodontic alignment. Am J Orthod Dentofac Orthop.

[25] Sarikaya S, Haydar B, Ciger S, Ariyurek M (2002). Changes in alveolar bone thickness due to retraction of anterior teeth. Am J Orthod Dentofac Orthop.

[26] Vardimon AD, Oren E, Ben-Bassat Y (1998). Cortical bone remodeling tooth movement ratio during maxillary incisor retraction with tip versus torque movements. Am J Orthod Dentofac Orthop.

[27] Ahn HW, Moon SC, Baek SH (2013). Morphometric evaluation of changes in the alveolar bone and roots of the maxillary anterior teeth before and after en masse retraction using cone-beam computed tomography. Angle Orthod.

[28] Thongudomporn U, Charoemratrote C, Jearapongpakorn S (2015). Changes of anterior maxillary alveolar bone thickness following incisor proclination and extrusion. Angle Orthod.

[29] Atik E, Gorucu-Coskuner H, Akarsu-Guven B, Taner T (2018). Evaluation of changes in the maxillary alveolar bone after incisor intrusion. Korean J Orthod..

[30] Lund H, Grondahl K, Grondahl HG (2012). Cone beam computed tomography evaluations of marginal alveolar bone before and after orthodontic treatment combined with premolar extractions. Eur J Oral Sci.

[31] Jamilian A, Gholami D, Toliat M, Safaeian S (2008). Changes in facial profile during orthodontic treatment with extraction of four first premolars [J]. Orthodontic Waves.

[32] Samandara A, Papageorgiou SN, Ioannidou-Marathiotou I, Kavvadia-Tsatala S, Papadopoulos MA (2019). Evaluation of orthodontically induced external root resorption following orthodontic treatment using cone beam computed tomography (CBCT): a systematic review and meta-analysis. Eur J Orthodont.

[33] Kaley J, Phillips C. Factors related to root Resorption in edgewise practice. Angle Orthod. 1991;61(2):125–32. 10.1043/0003-3219(1991)061<0125:FRTRRI>2.0.CO;2. 10.1043/0003-3219(1991)061<0125:FRTRRI>2.0.CO;2 2064070

[34] Horiuchi A, Hotokezaka H, Kobayashi K (1998). Correlation between, cortical plate proximity and apical root resorption. Am J Orthod Dentofac Orthop.

[35] Pan YC, Chen S (2019). Contact of the incisive canal and upper central incisors causing root resorption after retraction with orthodontic mini-implants: a CBCT study. Angle Orthod.

[36] Al-Abdwani R, Moles DR, Noar JH (2009). Change of incisor inclination effects on points a and B. Angle Orthod.

[37] Al-Nimri KS, Hazza'a AM, Al-Omari RM (2009). Maxillary incisor Proclination effect on the position of point a in class II division 2 malocclusion. Angle Orthod.

